# PreImplantation Factor (PIF) correlates with early mammalian embryo development-bovine and murine models

**DOI:** 10.1186/1477-7827-9-63

**Published:** 2011-05-15

**Authors:** Christopher W Stamatkin, Roumen G Roussev, Mike Stout, Victor Absalon-Medina, Sivakumar Ramu, Chelsi Goodman, Carolyn B Coulam, Robert O Gilbert, Robert A Godke, Eytan R Barnea

**Affiliations:** 1 BioIncept LLC/CARI Research Institute, Chicago, IL, USA; 2 Louisiana State University Embryo Biotechnology Laboratory, LSU Agricultural Center, Baton Rouge, LA USA; 3 Reproductive Medicine, Cornell University College of Veterinary Medicine, Ithaca, NY, USA; 4 SIEP, Society for the Investigation of Early Pregnancy, Cherry Hill, NJ, USA; 5 Department of Obstetrics, Gynecology and Reproduction, UMDNJ-Robert Wood Johnson Medical School, Camden, NJ, USA

## Abstract

**Background:**

PreImplantation Factor (PIF), a novel peptide secreted by viable embryos is essential for pregnancy: PIF modulates local immunity, promotes decidual pro-adhesion molecules and enhances trophoblast invasion. To determine the role of PIF in post-fertilization embryo development, we measured the peptide's concentration in the culture medium and tested endogenous PIF's potential trophic effects and direct interaction with the embryo.

**Methods:**

Determine PIF levels in culture medium of multiple mouse and single bovine embryos cultured up to the blastocyst stage using PIF-ELISA. Examine the inhibitory effects of anti-PIF-monoclonal antibody (mAb) added to medium on cultured mouse embryos development. Test FITC-PIF uptake by cultured bovine blastocysts using fluorescent microscopy.

**Results:**

PIF levels in mouse embryo culture medium significantly increased from the morula to the blastocyst stage (ANOVA, P = 0.01). In contrast, atretic embryos medium was similar to the medium only control. Detectable - though low - PIF levels were secreted already by 2-cell stage mouse embryos. In single bovine IVF-derived embryos, PIF levels in medium at day 3 of culture were higher than non-cleaving embryos (control) (P = 0.01) and at day 7 were higher than day 3 (P = 0.03). In non-cleaving embryos culture medium was similar to medium alone (control). Anti-PIF-mAb added to mouse embryo cultures lowered blastocyst formation rate 3-fold in a dose-dependent manner (2-way contingency table, multiple groups, X2; P = 0.01) as compared with non-specific mouse mAb, and medium alone, control. FITC-PIF was taken-up by cultured bovine blastocysts, but not by scrambled FITC-PIF (control).

**Conclusions:**

PIF is an early embryo viability marker that has a direct supportive role on embryo development in culture. PIF-ELISA use to assess IVF embryo quality prior to transfer is warranted. Overall, our data supports PIF's endogenous self sustaining role in embryo development and the utility of PIF- ELISA to detect viable embryos in a non-invasive manner.

## Background

The viable mammalian embryo controls its own destiny, transmitting specific signals to the mother/host throughout pregnancy [1]: Within the uterus, such signals support implantation [2] while in the periphery they induce and/or maintain tolerance without allowing for deleterious immune suppression to occur [3]. Evidently, acceptance signals by the mother also play an important role. Since the immune milieu of pregnancy is unique - not replicated in any other circumstances - specific embryo-derived signals have a crucial role leading to maternal recognition of pregnancy [4]. To orchestrate such critical 'cross-talk', a *viable *embryo must be present, which may be accepted by the mother, whereas, a non-viable conceptus will fail to develop or later be rejected since maternal acceptance does not occur. The search to identify embryo-specific markers which reflect viability of cultured embryos by assessing the medium or by performing an embryo biopsy (beyond morphological evaluation) has been ongoing. However, thus far no marker has entered into routine clinical use in humans or other mammals undergoing IVF procedures [5,6].

In humans, the platelet activating factor - (PAF) [7] and early pregnancy factor-(EPF) [8] have not been proven useful in selecting the best embryos for transfer to the recipient. Soluble HLA-G (sHLA-G) also failed to confirm its proposed utility in clinical studies [9]. Measurement of free radicals in the culture medium of developing embryos has been reported as an index for embryo viability. However, it is not an embryo-specific marker and the fate of the transferred embryo cannot be followed in maternal circulation [10].

The Barnea group reported that viable mouse and human embryos secrete a peptide, PreImplantation Factor (PIF) (MVRIKPGSANKPSDD), which is present in maternal circulation and is expressed by embryos and placental tissue [4,11-14]. In the placenta, PIF was found to be expressed in the trophoblastic layer in the first and second trimester while minimally being expressed at term as documented by staining using a specific anti-PIF-antibody [13]. Synthetic PIF analog (sPIF) replicates native PIF action, modulates peripheral immune cells to achieve tolerance without immune suppression, and has been demonstrated to be effective in autoimmunity models outside pregnancy [15-17]. PIF displays essential multi-targeted effects; regulating immunity, promoting embryo-decidual adhesion, and regulating adaptive apoptotic processes in cultured human decidual cells [18]. In addition, PIF promotes trophoblast invasion reflecting an autocrine supporting effect on conceptus development [19]. We have previously demonstrated that PIF is secreted by viable embryos [13]. Therefore, it is important to determine whether monitoring PIF levels in embryo culture medium could be of value in determining embryo developmental potential reaching to the blastocyst stage. Further, since PIF is an early secretory product, determining whether the peptide has embryo trophic effects could further substantiate its role in early pregnancy events.

Since access to large quantities of culture medium from human IVF embryos is limited (single embryos are cultured in low volumes), it was decided to examine PIF secretion into the medium using our ELISA methods in two different species offering complementary features. In mice, a large number of embryos can be cultured together which enables measurements of an expected low PIF level in the medium. Bovine embryos (larger size) can be successfully cultured singly to assess the source of PIF production by individual embryos. The single bovine embryo cultures serve as an IVF model where serial measurements of secreted products also can be determined.

The aims of the study were: 1. Determine PIF levels by ELISA in the medium of post-fertilization mouse embryos cultured up to the blastocyst stage. 2. Measure PIF levels by ELISA in the medium of cultured single bovine IVF embryos. 3. Test the inhibitory effect of anti-PIF-monoclonal antibody added to cultured mouse embryos. 4. Examine FITC-PIF uptake by cultured bovine blastocysts. We report that PIF is secreted by viable bovine and mouse embryos and we suggest that the peptide has a supporting role in embryo development.

## Methods

Biotin-conjugated mouse anti-PIF mAb, (proprietary of BioIncept LLC) horseradish peroxidase (HRP)-conjugated streptavidin (UltraAvidin-HRP), antigen-coating buffer and tetramethylbenzidine (TMB) substrate were all obtained from Leinco Technologies, Inc. (Saint Louis, MO). SEA BLOCK blocking buffer was obtained from Thermo Scientific (Waltham, MA). LumiGLO Peroxidase Chemiluminescent substrate was obtained from KPL, Inc. (Gaithersburg, MD). Synthetic PIF (MVRIKPGSANKPSDD) and scrambled PIF (GRVDPSNKSMPKDIA) (proprietary of BioIncept LLC) were obtained by solid-phase peptide synthesis (Peptide Synthesizer, Applied Biosystems, Foster City, CA) using Fmoc (9-fluorenylmethoxycarbonyl) chemistry. The same peptides were also labeled with FITC on their N-termini in the solid phase after the addition of l-alanine as a spacer group. Final purification was conducted by reversed-phase HPLC and identity was verified by MALDI-TOF mass spectrometry and amino acid analysis. Ovalbumin-conjugated PIF was also generated. (Biosynthesis, Inc., Lewisville, TX).

### Mouse embryo cultures

The study has been approved by Cari Research Institute. A routinely used mouse embryo culture procedure was used in this study [11,12]. 2-cell embryos were collected from super ovulated mated CB6F1/J mice. Removed oviducts were dissected under microscope and embryos removed in mHTF medium (n = 10 to110/well) and cultured in using 4 well (176740) and 24 well (142475) Nunc well plates in 500 μl droplets of culture medium (5% Quinns advantage blastocyst medium, RM-ART-1029 in 95% mHTF), Cat No 90125 (Irvine Bio, CA) and 10% FBS under mineral oil by incubating at 37°C with 5% CO_2 _for 3 days, at a pH 7.2 which was maintained throughout the culture period. In the first set of experiments, mouse embryos were cultured serially from the morula stage (15 dishes with 10 morula each) to the blastocyst stage (15 dishes with 10 blastocysts each) and PIF levels were compared with atretic embryos (those embryos that degenerated and failed to progress beyond the 2-cell stage). (8 dishes with 10 embryos each) or culture medium alone, used as control. In the second set of experiments to determine how early PIF is secreted, 2-cell stage embryos (102-110/well) were collected and cultured up to 4 hours and then the medium was collected. In the third set of experiments embryos (10 to15/group) were cultured in 100 μl droplets for 72 hours up to the blastocyst stage and PIF levels in the medium were determined and compared with medium alone (control). The data were calculated as estimated amount total in culture as well as estimated by individual embryos.

### *In vitro *fertilization procedure to retrieve bovine oocytes

The study has been approved by the Louisiana State University. At the Louisiana State University Embryo Biotechnology Laboratory, *in vitro *fertilization (IVF) was performed on bovine oocytes obtained from a commercial source (Ovitra, TX). Oocytes arrived in a climate controlled container via FedEx at ~24 hours following their collection. A standard bovine IVF laboratory procedure was performed on groups of 10 oocytes in 40 μl droplets of fertilization medium (IVF-TALP). Briefly, 2 μl of heparin (2 μg/ml), 2 μl of PHE and 2 μl sperm were added to each fertilization droplet with the M-II oocytes. This made the total medium volume 44 μl. Frozen-thawed sperm from a fertile Holstein bull was used in all bovine IVF experiments. The *in vitro *fertilization interval was 18 hours incubated in a humidified atmosphere of 5% CO_2 _in air at 39°C.

Following fertilization, the presumptive zygotes were removed from the fertilization droplets and treated with hyaluronidase (1 mg/ml) to remove the remaining cumulus cells. The embryos were then washed in HEPES-TALP medium and transferred to CR1aa medium [20]. A single embryo from the group of IVF-derived embryos was then placed into a fresh 40 μl droplet of CR1aa culture medium at 39°C in a humidified atmosphere of 5% CO_2 _in air. On day 3 of culture, individual embryos were transferred to a new 40 μl droplet of CR1aa and incubated at 39°C in a humidified atmosphere of 5% CO_2 _in air and were cultured in the same medium until day 7 of culture. At the end of the experiment, embryos were assigned an embryo quality grade (1 = good to 3 = poor) and evaluated for morphological development (2-cell stage through to the blastocyst stage).

### PIF secretion in IVF-derived bovine embryos during *in vitro *culture

IVF was performed on M-II bovine oocytes (n = 116). Following fertilization, single normal appearing zygotes were then placed into an individual 40 μl droplet of CR1aa culture medium [21] (n = 120). On day 3 of culture, embryos were transferred to a new 40 μl droplet of CR1aa medium for days 3 to 7 of *in vitro *culture. Only embryos that showed morphological development were transferred to fresh individual culture droplets. Morphological development of the embryos on days 1, 3 and 7 was recorded just prior to being moved to a fresh culture medium. After the embryos were moved from the 40 μl droplets, two 10 μl samples of conditioned culture medium from day 3 and day 7 culture were collected and frozen for subsequent determination of PIF concentrations using a PIF- ELISA described below.

### Neutralize endogenous PIF using anti-PIF monoclonal antibody

An anti-PIF monoclonal antibody was developed (Genway, Inc. San Diego, CA.) and its specificity was subsequently validated (see ELISA methods). Mouse 2-cell stage embryos (n = 19-36/group) were cultured with increasing antibody concentrations (0.047-47 μg/ml). After culturing for 72 hours, the number of embryos that developed at each morphologic stage was recorded. Embryo quality and *in vitro *survival were evaluated from the 2-cell stage through the blastocyst stage. Also, the number of atretic embryos following exposure to each treatment was noted. These results were compared with exposure to the non-specific mouse anti-IgG isotype monoclonal antibody (Zymed, San Francisco, CA) that served as the negative control and culture medium alone that served as an additional control.

### PIF-ELISA methods: sandwich PIF-ELISA

Affinity purified anti-PIF Ab (rabbit IgG, Covance Laboratories, PA) in bicarbonate buffer (pH 9.0) was diluted 1:2000 and 100 μl of the solution was added to 96 -well plates, incubated for 1 hour at 37°C and then refrigerated overnight at 4°C, then washed three times with PBS. Each plate was blocked with 100 μl 0.2% of nonfat dry milk in PBS and incubated for one hour at room temperature followed by washing with PBS. Either 50 μl mouse embryo culture medium or 50 μl PIF standards (0.007 to 1 μg/ml) was added to plates. Following 1 hour incubation, plates were washed with PBS. Then 100 μl of anti-PIF monoclonal antibody at 1:4000 in PBS was added and incubated for 1 hour followed by washing with PBS. 100 μl conjugated goat anti-mouse IgG alkaline phosphatase (Pierce, Rockford, IL) was added and incubated for 1 hour followed by washing with PBS. 100 μl substrate buffer (p-nitrophenyl) (Pierce) was added and the reaction was stopped after 15-20 minutes with 70 μl 3M NaOH. A standard curve is shown (see Additional file 1, Figure S1). Also the anti-PIF-monoclonal antibody properties that are provided comparing PIF binding as compared with scrambled PIF (no binding) (see Additional file 2, Figure S2). The affinity of anti-PIF-monoclonal antibody to PIF is shown (see Additional file 3, Figure S3). The serial mouse embryo culture data was generated with the sandwich assay.

### Chemilluminescent assay

**Figure 1:**
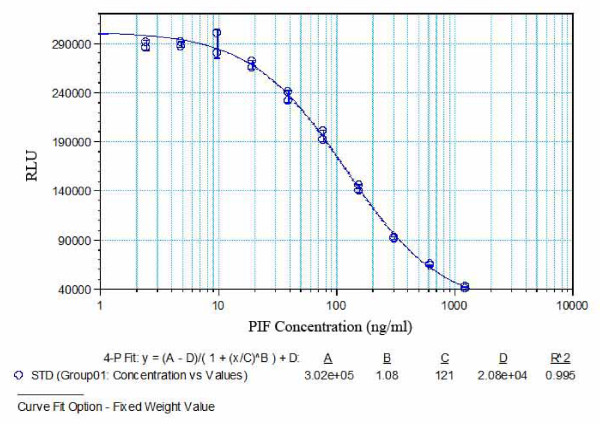
**PIF detection in viable embryo culture medium using anti-PIF-mAb-based chemiluminescent ELISA**. Representative standard curve of PIF ELISA demonstrates that low levels of the peptide can be detected. The assay was linear where 2 ng/ml was the lowest detection limit.

Briefly, ovalbumin-PIF was diluted in Ultracoat II buffer (100 ng/ml) and 100 μl was added to each well of 96 well-plate, incubated overnight, washed and blocked with 300 μl of SEA BLOCK buffer for 2 hours at 37°C, then washed and air dried. Both mouse and bovine embryo culture medium were diluted in PBS, 1:2 or with duplicate sPIF standards (1to1,250 ng/ml) and 50 μl of the medium was added to 50 μl of biotin-anti-PIF mAb solution, (1:20,000), except for the blanks. Following incubation for 2 hours, wells were washed, 100 μl UltraAvidin-HRP was added and incubated for 30 minutes at 37°C. After washing, 100 μl LumiGLO was added to each well for 5 minutes and plates were read in a SpectraMax L microplate luminometer and analyzed using SoftMax Pro software (Molecular Devices, Sunnyvale, CA). Assay linearity and anti-PIF-monoclonal antibody specificity are shown (Figure 1). Background assay levels were 10 ng/ml tested with several different serum-free embryo culture medium also used in the present study. Assay readings were linear down to 2 ng/ml. Spike and recovery experiments at 30 ng/ml and 70 ng/ml confirmed recovery at 95% and 115%, respectively. The early mouse embryo and blastocyst data as well as all the bovine embryo data was generated using the chemilluminescent ELISA.

### Studies for FITC-PIF uptake by bovine blastocysts: Oocyte recovery and selection

The study has been approved by Cornell University College of Veterinary Medicine. Ovine ovaries were collected at nearby abattoir and transported to the laboratory in prewarmed lactated Ringer's solution at 30-35°C. Cumulus oocyte-complexes (COCs) from 2-8 mm follicles were aspirated with an 18G hypodermic needle attached to an aspiration pump unit adjusted to a pressure of 22.5 ± 2.5 ml of H_2_O per minute. Follicular fluid supernatant was removed and the pellet containing COCs was transferred to a 15 ml tube where it was resuspended with holding media and contents were poured gently into a 100 mm Petri dish. Holding media consisted on TCM-199 Hank's salts (Invitrogen []) 10% Fetal Calf Serum (Invitrogen), 25 μg/ml gentamicin and heparin 4 μg/ml (sigma) with a mOsm = 300 ± 1. Morphological selection of COCs consisted of using only those showing several layers of granulosal cumulus cells and oocytes with a homogenous cytoplasm.

### *In vitro *maturation

Selected COCs were matured in groups of 40 ± 5 for 23 ± 1 hours in 400 μL of TCM-199 (Earle's Salts [Sigma M2154]) enriched with 10% fetal calf serum (FCS; Invitrogen), 22 μg/ml sodium pyruvate, 1 mM alanyl-glutamine, 0.1 mM taurine, 0.1 mM cysteamine, 1 μg/ml estradiol, 5 μg/ml luteinizing hormone (LH; SIOUX Biochemical, Inc., Sioux Center, IA), 0.5 μg/ml follicle stimulating hormone (FSH; SIOUX), 10 ng/ml epidermal growth factor (EGF; BD Biosciences--Discovery Labware, MA), 25 μg/ml gentamicin, pH 7.35 ± 2 and mOsm 300 ± 2 covered with mineral oil in a humidified atmosphere at 38.5°C with 5% CO_2 _in air.

### *In vitro *fertilization

After a total of 23 ± 1 hours presumptive matured oocytes were transferred to a modified IVF medium (Fert-TALP) [20] supplemented with 0.5 mM fructose, 6 mg/ml BSA FFA Fraction V, 30 μM penicilamine, 15 μM hypotaurine, 1.5 μM epinephrine (PHE), 22 μg/ml heparin, covered with mineral oil in a humidified atmosphere at 38.5°C with 5% CO_2 _in air for 18-22 hours (pH of 7.38 ± 1, mOsm 290 ± 2). Frozen semen samples from a single bull were thawed and sperm was separated from the seminal plasma and cryoprotectant by percoll step gradient (90, 45%) centrifugation at 700 × g for 20 min. Subsequently, sperm was washed twice in 5 ml of TL-Semen (pH 7.39 ± 1, mOsm 295 ± 2) at 300 × g for 5 min to remove Percoll. Finally, sperm was adjusted to a final concentration of 1.5 × 10^6 ^sp/ml and with an average progressive motility of 50% using Fert-TALP media.

### *In vitro *embryo culture

A modified synthetic oviductal fluid (SOF) [22] media was used for the *in vitro *embryo culture (pH 7.4 ± 1, mOsm 275 ± 5). After fertilization, putative zygotes were denuded at maximum vortex for 120 seconds and transferred to a modified SOF (SOF early) supplemented with 10 μM EDTA, 0.5 mM fructose, 4 mg/ml BSA FFA Fraction V, 0.1 mM taurine, and without essential amino acids covered with mineral oil in a humidified atmosphere at 38.5°C with 5% CO_2_, 5% O_2_, and 90% N_2 _in air for ~48 hours. After that time, cleavage rates were assessed and embryos were transferred to new droplets containing SOF mid, which was supplemented with essential and non-essential amino acids, 4 mg/ml BSA FFA Fraction V, and 0.5 mM glucose in a humidified atmosphere at 38.5°C with 5% CO_2_, 5% O_2_, and 90% N_2 _in air for ~96 hours. Embryos were transferred to fresh SOF mid droplets under the same conditions after the first ~48 hours. Finally, d-7 embryos were transferred for the last ~24 hours of culture to SOF late, which was SOF supplemented with 5% fetal calf serum (FCS), 0.1 mM taurine and 0.5 mM glucose in a humidified atmosphere at 38.5°C with 5% CO_2_, and 5% O_2_, and 90% N_2 _in air. Further, blastocyst rates were based on percentages of the original oocyte number.

### FITC-PIF uptake by bovine blastocysts

After eight days post-fertilization, embryos were sorted by stage (i.e. blastocyst, expanded blastocyst and hatched blastocyst) (N = 23) and cultured for 30 minutes in synthetic oviductal media droplets containing 5 μg/ml FITC-PIF or FITC-PIFscrambled in a humidified atmosphere, 5% CO_2_, 5% O_2 _and balanced N_2 _with a temperature 38.5°C. Blastocysts were counterstained with DAPI (diamidino-2-phenylindole) a blue fluorescent probe that fluoresces brightly upon selectively binding to the minor groove of double stranded DNA, (nuclear) where its fluorescence is approximately 20-fold greater than in the non-bound state. Subsequently, blastocysts were washed in PBS/PVP solution to remove non-specific binding and fixed in 10% formalin containing 0.1% triton x-100. Fixed embryos were mounted on slides with two etched 10 mm diameter circles surrounded by white ceramic ink, embryos were covered with glycerol containing Hoechst 33348 (3 μg/ml).

### Image acquisition

All slides were visualized using a microscope (Imager Z1; Carl Zeiss, Inc.) under a 20 × 0.5 NA ECPlan Neofluar air immersion (Carl Zeiss, Inc.). The fluorochromes used FITC and nuclei were observed using DAPI filter. Slides were excited at 340 nm and 488 nm to visualize DAPI nuclear stain and FITC, respectively. Images were captured with a cooled charged-coupled device camera (AxioCam MRm; Carl Zeiss, Inc.) and processed using AxioVision software (version 4.7.2; Carl Zeiss, Inc.). Images untouched were taken as superimposed (FITC/DAPI), FITC and DAPI, alone.

### Statistical Analysis

PIF secretion by mouse embryo cultures and by cow embryos was analyzed by ANOVA followed by paired Student's t-test. The proportion of mice embryos that were affected by exposure to anti-PIF monoclonal antibody was determined using a 2-way contingency multi-group Chi square analysis. Analysis was carried out using Analyse-it for Microsoft Excel. A P < 0.05 level was considered to be significantly different in these studies.

## Results

### PIF is secreted by viable embryos

**Figure 2:**
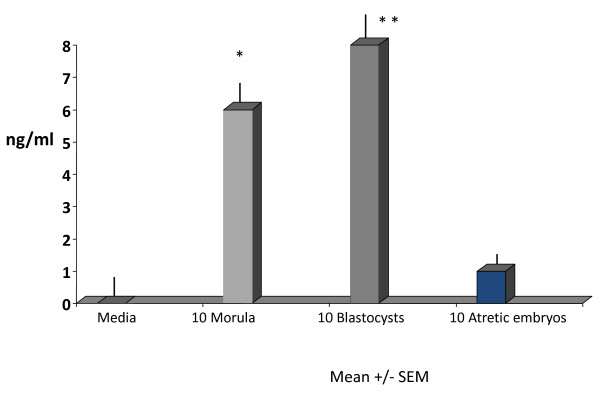
**PIF detection in culture medium was associated with mouse embryo viability**. Mouse embryos were cultured in groups up to the morula stage medium collected and further cultured up the blastocyst stage collecting the medium. PIF levels were compared with cultured atretic embryos and medium control. Data are expressed mean+/-SEM. PIF levels were significantly higher in both morulae and blastocysts (p = 0.05 and p = 0.01) respectively, as compared to similar levels obtained in atretic and medium alone used as control.

The dynamics of PIF secretion by cultured mouse embryos was assessed. We showed that PIF was detected in the medium of morula stage embryos cultured in groups and PIF levels further increased in the blastocyst stage in serially cultured embryos (ANOVA, P = 0.05 and P = 0.01), respectively as compared with controls. (Figure 2). In contrast, PIF secretion by atretic embryos was minimal with values that were similar to medium alone. Thus, increased PIF levels reflect progressive embryo development.

### PIF secretion starts at the 2-cell stage

How early post-fertilization is PIF secreted? Two-cell stage mouse embryos (n = 102-110) were cultured *in vitro *for only 4 hours. Measurable (but low) PIF levels were detected by using our sensitive chemiluminescent ELISA (Table 1). In embryos cultured up to the blastocyst stage, (10-15/group) PIF levels were higher. Per embryo, PIF appeared to be ~10-12 fold higher in blastocysts than in 2-cell embryos. Hence, PIF is a secreted product of early stage embryos and levels are higher at a later developmental stage.

**Table 1:** PIF secretion by mouse embryos at day 0 and day 3 of culture

Day 0	Day 3
3.2	26.2
56.4	35.4
23.1	83.9
9.5	33.6
	34.5
	50.1
	62.6
Mean +/-SEM 23.7+/-13.5	46.6+/-7.7

Mouse embryos were cultured at 2-cell stage for 4 hours (N = 102-110/embryos) and the media was collected for analysis from 4 different culture media and analyzed for PIF levels. Also, mouse embryos were cultured at 2-cell stage (N = 10-15/embryos) for 72 hours and the media was collected for PIF analysis from 7 different cultures. Results are mean values+/- SEM of results of independent cultures. Chemiluminescent ELISA. Background levels were substracted from each experiment. Data is expressed as ng/ml.

### PIF is secreted by viable individual IVF derived embryos (bovine)

**Figure 3:**
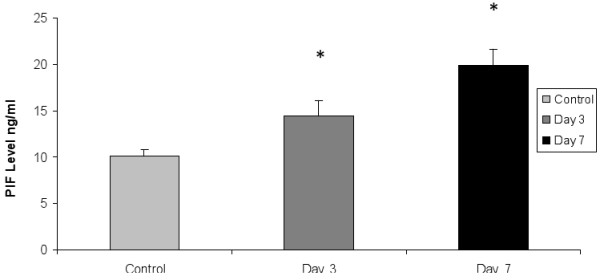
**PIF detection in single cultured bovine IVF embryos medium. PIF levels increase in viable embryos**. Cleaving embryos were cultured from day 3 to day 7 reaching up to the blastocyst stage. PIF levels Mean+/- SEM in the medium were significantly higher compared with levels in non-cleaving embryos (ANOVA followed by student t-test): Control (medium alone, or medium of non-cleaving embryos (n = 15) vs. day 3 P = 0.01, Control *vs*. day 7, P = 0.005, Day 3 *vs*. day 7, P = 0.03).

**Figure 4:**
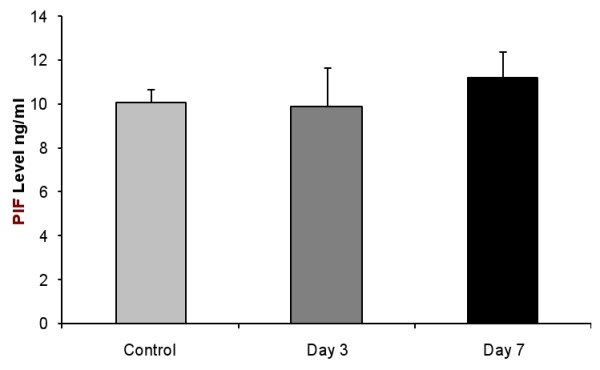
**PIF levels in atretic embryos are similar to non-cleaving embryos**. The medium collected from embryos that failed to progress from day 3 to day 7 of culture were analyzed sequentially and levels Mean+/-SEM were compared with non-cleaving embryos. There were no significant differences among the groups tested (n = 5-15/group).

**Figure 5:**
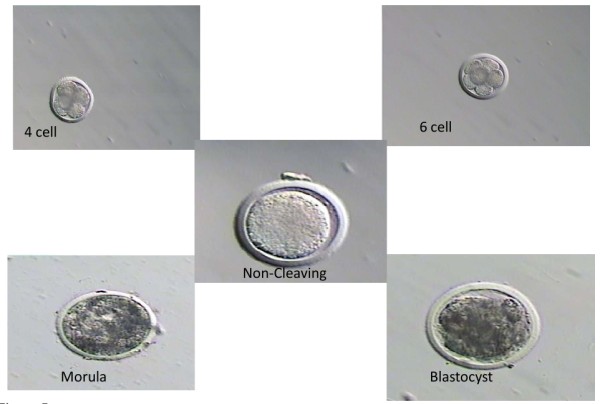
**Microphotographs of individually cultured viable and non-viable bovine IVF embryos**. It shows a representative group of bovine embryos cultured from the 6-cell to blastocyst stage, which were used to determine PIF levels in their culture medium as well a non- cleaving embryo, control.

We examined the pattern of PIF secretion by individual IVF derived bovine embryos in culture. It is recognized that in the bovine species cultured, single embryos frequently do not progress to the blastocyst stage and many embryos will fail to cleave. For analysis we used PIF values (Mean+/-SEM) of developing embryos comparing the levels in the same embryos at day 3 to levels present at day 7. Those embryos that degenerate cultured from day 3 to 7, showing no change, and those that fail to cleave used as control. We found that PIF levels increased significantly from day 3 to day 7 in the culture medium, and in developing single embryo cultures (ANOVA, P = 0.03). The difference between day 3 cleaving embryos and the control (non-cleaving embryos, or medium used as background control) at day 3 of culture were significantly different (P = 0.01). In all embryos, a microphotograph was taken to compare the morphology to PIF levels (Figure 3). Individual embryo's PIF levels ng/ml at day 3 were as follows: 6 cells (10.7), 8 cells (12.6-18.2), day 7; 32 cells (16.3) morula (13.3-26.8) and blastocyst (21.3). PIF was already detected at the 8 cells stage and in all viable embryo culture media up to the blastocyst stage. All PIF levels in viable embryos were significantly higher than medium only control. In contrast, PIF concentration in medium of cow embryos that failed to develop at day 3 (9.7+/-1.8) (N = 5) or even at day 7 (11.2+/- 1.4 (N = 5) were similar to levels in medium of non-cleaving embryos or media alone (background levels (control) Mean+/- SEM 10+/-1 (N = 15)), (P = 0.87, NS). (Figure 4) Microphotographs of representative cow embryos are shown (N = ~100) (Figure 5). Therefore, increasing levels of PIF in cultured IVF bovine embryos correlate with their development.

### Endogenous PIF is a requirement for early embryo development

Since PIF is secreted shortly post-fertilization (2-cell stage) in mice, we examined endogenous peptide's potential role in controlling mouse embryos development. Addition of anti-PIF antibody to cultures significantly reduced mouse blastocyst formation rates as compared with the total number of cultured embryos (Table 2). All anti-PIF-mAb doses tested reduced blastocyst formation and increased rates of atretic and 2-cell embryos as compared with the controls 2-way contingency table, multiple groups, (X^2^;P = 0.01). At the highest anti-PIF-mAb concentration (47000 ng/ml) blastocyst rates were only 21% while atretic embryo rates were elevated 63%. The effect was dose-dependent; at the lowest antibody concentration 47 ng/ml blastocyst rates were higher (41%) and atretic rates were lower (34%) as compared with the highest antibody concentration tested (X^2^, P = 0.03). In contrast, in both tested control groups the non-specific mouse antibody and medium control blastocyst rates were significantly higher than any anti-PIF antibody concentration tested 77% and 68%, respectively. Also no atretic embryos were observed in the two control groups (X^2^, P = 0.01, multigroup). Hence, endogenous PIF is required to support optimal embryo (blastocyst) development in the mouse.

**Table 2:** Anti-PIF-antibody reduces mouse embryos blastocyst development

Anti-PIF Mab Titration N =		24		26		36		27		30		19
	47000 ng/mL	%	4700 ng/mL	%	470 ng/mL	%	47 ng/mL	%	**Neg. Contr**.	%	**Isotype Contr**.	%
Blastocyst	5	0.21	8	0.31	11	0.32	11	0.41	23	0.77	13	0.68
Eblast	2	0.08	2	0.08	7	0.21	4	0.15	5	0.17	3	0.16
Morula	2	0.08	0	0.00	4	0.12	0	0.00	2	0.07	2	0.11
6-8 cell	0	0.00	7	0.27	2	0.06	3	0.11	0	0.00	0	0.00
2-4 cell	11	0.46	5	0.19	11	0.32	8	0.30	0	0.00	1	0.05
AT > Morula	0	0.00	2	0.08	0	0.00	0	0.00	0	0.00	0	0.00
AT < Morula	4	0.17	2	0.08	1	0.03	1	0.04	0	0.00	0	0.00

Mouse 2cell embryos were collected from mice and cultured with increasing concentrations of anti-PIF-antibody added to the culture media. (N = 19-36/group). Results were compared with isotype antibody and medium alone used as controls. Data is expressed as number of embryos in each developmental stage. Inhibitory effect of anti-PIF-antibody was dose dependent. Data analyzed by using (2-way contingency table, multiple groups, (X^2^; P = 0.01).

### FITC-PIF is being uptaken by cow blastocysts

**Figure 6:**
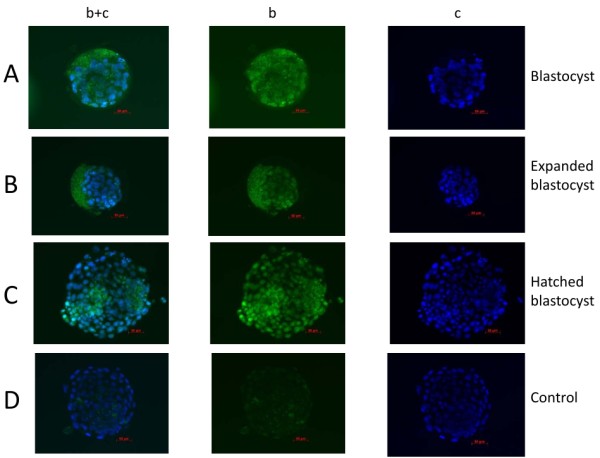
**FITC-PIF is selectively taken up by bovine blastocysts**. Blastocysts were exposed to either 5 μg/ml FITC-PIF or scrambled FITC-PIF for 30 minutes in culture and specific binding was evaluated by washing off unattached fluorescent peptide. Blastocysts nuclei were also counterstained using DAPI, vital stain. Embryos were fixed, stained and evaluated by using a fluorescent microscope using two different wave lengths. b+c (FITC-PIF or scrambled FITC-PIF and DAPI stain), b, (FITC-PIF or scrambled FITC-PIF alone), c (DAPI stain alone). Part A: FITC-PIF binds to cultured blastocysts. Part B: FITC-PIF binds to expanded blastocysts. Part C: FITC-PIF binds to hatched blastocysts Part D. In contrast, 5 μg/ml FITC-scrambled PIF, used as control did not bind. (magnification × 40).

Since PIF secretion correlated with cow embryo development. We aimed to examine whether PIF also being uptaken by those embryos in culture. We found that after culturing blastocysts with FITC-PIF significant uptake was noted (Figure 6A, B, C, D). The binding was documented in blastocysts, expanded blastocysts and hatched blastocysts. In contrast, scrambled FITC-PIF, failed to bind (control). However, the FITC-PIF uptake/staining was not specific for the ICM/TB cells as all cells appeared to be labeled.

## Discussion

PreImplantation Factor is secreted by viable embryos (absent in non-viable embryos) and has been reported to play an essential role in early pregnancy events: creating maternal tolerance and promoting embryo implantation starting shortly post-fertilization [18,19]. We demonstrate herein that PIF is secreted by both viable mouse and bovine IVF embryos, indicating that the peptide may serve as a universal embryo viability marker. PIF secretion starts shortly post-fertilization, detected as early as the 2-cell stage, and reflects PIF's critical role in the earliest stages of mammalian embryo development. Neutralization of endogenous PIF effect reveals that the peptide is needed for embryos to progress to the blastocyst stage, since PIF exerts an obligatory direct supportive role. Hence, PIF-ELISA is an effective method to assess non-invasively the viability of cultured embryos at the earliest developmental stages and correlate PIF levels with their potential reaching the blastocyst stage. PIF-ELISA may thus represent a reliable tool to facilitate IVF embryo selection for transfer into the maternal host.

PIF was already detected at the 2-cell stage in mouse embryo culture medium. This confirms our previous results on PIF activity in both mouse and human embryo culture medium [14,23]. However, as expected given the limited secretion by mouse embryos, PIF detection required large group of embryos to be cultured together. These results confirm the intimate involvement of PIF in embryo development since levels are higher in cultured blastocysts, as compared with morula. Thus, the increased peptide levels correlated with a more advanced developmental stage. In contrast, PIF was not detected in atretic embryo culture medium. Given the inherent murine model limitations, multiple embryos (due to low-level PIF production) were required to be cultured; which did not permit to establish individual embryos contribution of PIF secreted into the medium. To overcome this limitation, culturing single bovine IVF embryos enabled to directly correlate PIF levels (within the same embryo) with development up to the blastocyst stage. As expected, in failed bovine embryos, (those that degenerate), levels were similar to non-cleaving embryos alone (assay background levels).

From these findings, PIF emerges as an early, reliable, and non-invasive embryo-specific viability marker which could improve IVF success rates without the need to culture embryos up to the blastocyst stage before transfer to the maternal host. This is likely since in the models used, PIF levels in embryos were determined in sequential cultures. Thus, early detection of PIF predicted a further progress in embryo development. PIF-ELISA method is easy to implement and results are available in <3 hours. And the assay is highly specific since the antibody does not cross-react with scrambled PIF, and the monoclonal antibody is highly potent.

We view that the data from single bovine embryo culture provides the evidence that PIF secretion increases with developmental stage, and therefore implementation for human IVF setting may be valuable. Thereby, PIF-based testing could be a valuable marker for assessing embryo viability in the medium, reducing the need for multi-embryo transfer. Preliminary evidence in that respect has been recently generated in women using an anti-PIF antibody-based immunoassay where detection in singly cultured embryo culture medium correlated with live birth post-multiple embryo transfer. Also more recently, no pregnancy has resulted following the transfer of single PIF negative embryos in women following IVF [23,24].

Since PIF is already secreted by the post-fertilization embryo, the possible role of the peptide in modulating its own development was also examined. It was found that endogenous PIF levels are directly associated with embryo viability since adding of anti-PIF-mAb to the medium at the 2-cell stage markedly inhibited mouse embryo development, leading most frequently to their demise. Therefore, embryo-secreted PIF likely exerts an supportive role on embryo development starting shortly post-fertilization.

Hence, in our view the embryo emerges as a semi-autonomic entity that operates within the maternal milieu and PIF plays an essential role in promoting this relative independence. This is especially relevant during the period when the embryo is surrounded by the zona pellucida that may limit the access of trophic agents from the maternal host. Since the expression of the majority of embryo-derived compounds is lower post-fertilization, it is remarkable that PIF would be secreted shortly post-fertilization and its expression will continue also throughout pregnancy [12]. The use of a non-specific monoclonal antibody as negative control that failed to affect embryo survival, verified that the anti-PIF-mAb effect was specific.

The anti-PIF-mAb data raises the question: how does PIF exert its embryo-trophic effect and whether the effect is direct or indirect. We found that FITC-PIF is being taken-up by the cultured cow blastocyst, which implies the presence of an autocrine loop. This might also explain the data generated using anti-PIF-mAb to inhibit embryo development. Where a break in this autocrine loop (the secreted PIF is not being taken-up) has led to the arrested embryo development. The identification of endogenous sites within the embryo where PIF interacts is an active area of investigation. To document binding specificity, two different stains were performed. The DAPI stain documented that cells were intact and nucleus is well preserved. Then images were superimposed with FITC-PIF staining. The data showed progressive change in location of FITC-PIF as the blastocyst has expanded (polarization?). The finding that the labeled scrambled PIF failed to bind reflects PIF specificity. In addition, similar observations using FITC-PIF uptake in culture mouse blastocysts were observed where a similar polarization was observed. (unpublished). This suggests that in both species an autocrine loop is present, which may have a role in supporting embryo development shortly post-fertilization. Support for PIF's autocrine trophic role was recently documented in singly cultured bovine embryos when addition of sPIF for the first three days increased the number of embryos reaching up to the blastocyst stage at day 7 [25].

This study is limited although FITC-PIF binding to blastocysts was demonstrated, the specific sites of interaction were not precisely identified. Strengths of the research are the use of a specific sensitive monoclonal antibody-based PIF-ELISA that enables detection of low PIF levels and the use of two different mammalian species for embryo culture evaluation.

Previously, it was reported that culturing embryos in groups promote their development through a possible "cross talk". In addition, other possible supportive signals in embryos have been reported [26-30]. Our work clearly suggests that PIF has an important autocrine trophic cross-talk role.

## Conclusions

In conclusion, endogenous PIF is required for embryo development and the supportive effects of the peptide are exerted directly on the embryo. Our observations add a novel facet for PIF secreted by the post-fertilization embryos; complementing the peptide's global role. Whereby, the embryo is controlling its own destiny by creating a favorable environment to enhance its development. PIF-ELISA is established as a valuable tool for PIF detection in culture medium of mammalian embryos reflecting on and correlating with their viability and progress to later stages of development and possible subsequent pregnancy success. The use of PIF measurements to improve embryo selection for transfer post-IVF is warranted and is currently being implemented in multicenter studies.

## Competing interests

PIF is a proprietary compound owned by BioIncept, LLC, (Biotech start-up) which holds several U.S. and foreign patents. PIF was discovered by ERB, who is the Company's (uncompensated) Chief Scientist. CBC owns 4% of the shares of BioIncept LLC. CWS, RGR, CG, received part of the funding for their compensation, during the period when the relevant studies were conducted, through a grant from BioIncept LLC to CARI. RAG and MS, VAM, and ROG declare no conflicting interest. JHB, President and CEO of BioIncept, LLC owns a majority of remaining shares.

## Authors' contributions

CWS and RGR conducted the mouse embryo experiments. CWS, SR helped ERB to develop the PIF ELISA and CBC provided the medium and CG tested and analyzed the culture medium. MS, RAG, provided single IVF embryos and carried out the bovine embryo cultures. RG and VA carried out the culture and FITC-PIF staining of cow blastocysts. ERB discovered PIF, developed the PIF technology, analyzed the data and wrote the manuscript. All authors read and approved the final manuscript.

## Supplementary Material

Additional file 1 **Figure S1**. Sandwich ELISA STD. Click here for file

Additional file 2 **Figure S2**. Comparison Anti-PIF-IgG binding to PIF vs PIFscr. Click here for file

Additional file 3 **Figure S3**. Anti-PIF- monoclonal antibody standard curve. Click here for file
