# Supplementary material for: PreImplantation Factor (PIF) correlates with early mammalian embryo development-bovine and murine models
Source: Reprod Biol Endocrinol. 2011 May 15;9:63. doi: 10.1186/1477-7827-9-63 (PMC3112407; doi:10.1186/1477-7827-9-63)
Supplement: Additional file 1 — Figure S1. Sandwich ELISA STD. [file 1477-7827-9-63-S1.PDF]

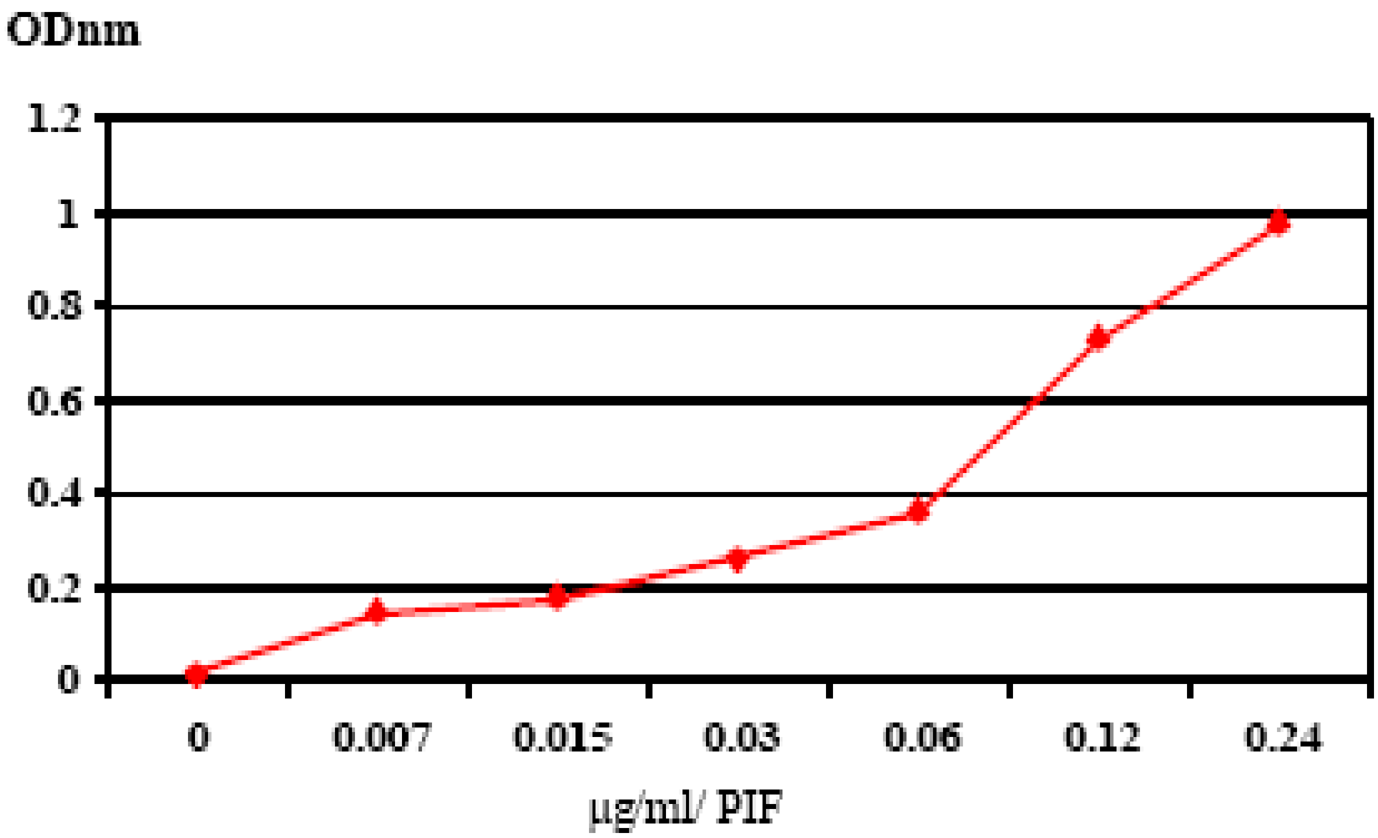

Sandwich ELISA STD. A standard curve was generated by using different concentrations of sPIF. The ELISA method was described in the methods section.

Figure S1
