# Supplementary material for: PreImplantation Factor (PIF) correlates with early mammalian embryo development-bovine and murine models
Source: Reprod Biol Endocrinol. 2011 May 15;9:63. doi: 10.1186/1477-7827-9-63 (PMC3112407; doi:10.1186/1477-7827-9-63)
Supplement: Additional file 2 — Figure S2. Comparison Anti-PIF-IgG binding to PIF vs PIFscr. [file 1477-7827-9-63-S2.PDF]

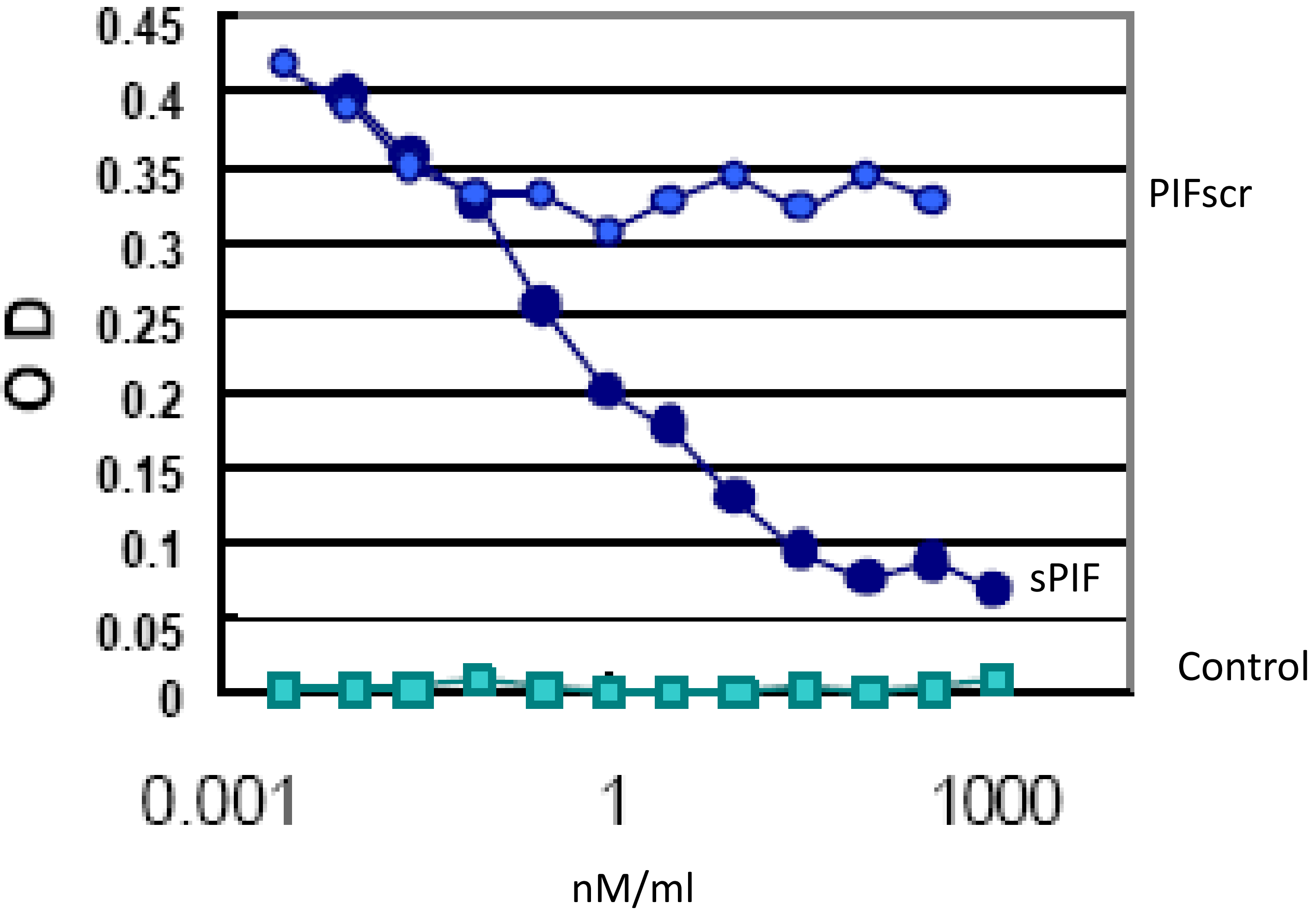

Comparison Anti-PIF-IgG binding to PIF vs PIFscr. Affinity purified anti-PIF-IgG was tested against 4.5 μg/ml sPIF significant binding was found at 10-30 pM IC at 500-700 pM. Linearity up to 30nM vs. PIFscr which did not compete Biotin-PIF.

Figure S2
