# Supplementary material for: PreImplantation Factor (PIF) correlates with early mammalian embryo development-bovine and murine models
Source: Reprod Biol Endocrinol. 2011 May 15;9:63. doi: 10.1186/1477-7827-9-63 (PMC3112407; doi:10.1186/1477-7827-9-63)
Supplement: Additional file 3 — Figure S3. Anti-PIF- monoclonal antibody standard curve. [file 1477-7827-9-63-S3.PDF]

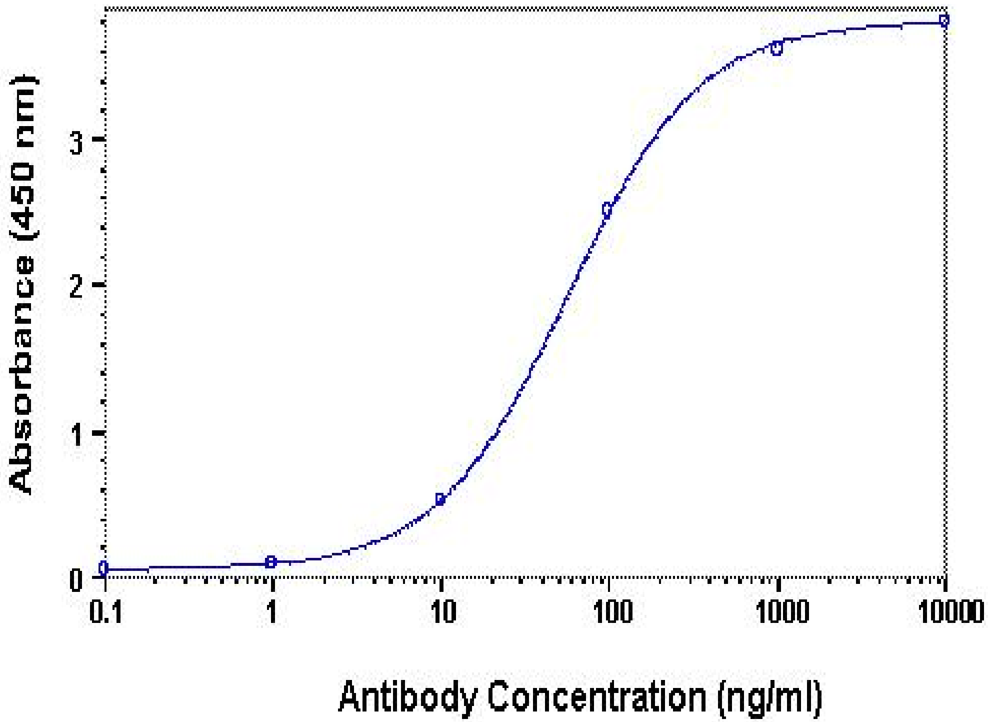

Anti-PIF- monoclonal antibody standard curve. Mouse monoclonal IgG was as primary antibody and Goat anti-Mouse IgG-HRP as 2nd antibody. Fixed amount of antigen (5 ug/ml) and serial dilutions of anti-PIF-Mab. The assay was linear up to 10ng/ml antibody.

Figure S3
